# Investigation of the Microstructure Evolution and Deformation Mechanisms of a Mg-Zn-Zr-RE Twin-Roll-Cast Magnesium Sheet by In-Situ Experimental Techniques

**DOI:** 10.3390/ma11020200

**Published:** 2018-01-27

**Authors:** Kristián Máthis, Klaudia Horváth, Gergely Farkas, Heeman Choe, Kwang Seon Shin, Alexei Vinogradov

**Affiliations:** 1 Department of Physics of Materials, Charles University, Ke Karlovu 5, 12116 Prague 2, Czech Republic; mathis@met.mff.cuni.cz (K.M.); farkasgr@gmail.com (G.F.); 2 Nuclear Physics Institute of the CAS, 250 68 Řež, Czech Republic; 3 School of Materials Science and Engineering, Kookmin University, 77 Jeongneung-ro, Seongbuk-gu, Seoul 136-702, Korea; heeman@kookmin.ac.kr; 4 Department of Materials Science and Engineering, Research Institute of Advanced Materials, Seoul National University, Seoul 08826, Korea; ksshin@snu.ac.kr; 5 Department of Mechanical and Industrial Engineering, Norwegian University of Science and Technology—NTNU, 7491 Trondheim, Norway; alexei.vinogradov@ntnu.no; 6 Institute of Advanced Technologies, Togliatti State University, 445020 Togliatti, Russia

**Keywords:** magnesium, twin roll casting, neutron diffraction, acoustic emission, twinning, yield strength

## Abstract

Twin roll casting (TRC), with a relatively fast solidification rate, is an excellent production method with promising potential for producing wrought semi or final Mg alloy products that can often suffer from poor formability. We investigate in this study the effect of the TRC method and the subsequent heat treatment on the microstructure and deformation mechanisms in Mg-Zn-Zr-Nd alloy deformed at room temperature using the in-situ neutron diffraction and acoustic emission techniques and ex-situ texture measurement and microscopy, respectively. Although a higher work hardening is observed in the rolling direction due to the more intensive <*a*>-type dislocation activity, the difference in the mechanical properties of the specimens deformed in the RD and TD directions is small in the as-rolled condition. An additional heat treatment results in recrystallization and significant anisotropy in the deformation. Due to the easier activation of the extension twinning in the TD given by texture, the yield stress in the TD is approximately 40% lower than that in the RD.

## 1. Introduction

Nowadays, achieving fuel saving through weight reduction of vehicles is a key task in the transportation industry. Thus, magnesium alloys, being among the lightest structural materials exhibiting an outstanding specific strength, receive a great deal of attention. Magnesium, owing to its hexagonal closed packed structure (hcp), exhibits a low strength and ductility at ambient temperatures. Therefore, various alloying elements, including zinc (Zn), zirconium (Zr) and/or different rare earth (RE) elements, are added for enhancing the mechanical performance [1]. Furthermore, the formability of magnesium alloys can be improved through microstructural modifications of the material by deformation processing, e.g., extrusion or rolling, at elevated temperatures stimulating activation of non-basal slips and the occurrence of dynamic recrystallization.

Twin roll casting (TRC) is an established cost-effective production method offering the possibility to fabricate wrought Mg (semi-) products for many weight-sensitive applications. The relatively fast solidification rate of the alloy during TRC has beneficial effects on the resultant microstructure such as reducing segregation, refining microstructural features, attaining higher homogeneity and extending solid solubility [2]. It enables better utilization of alloying elements having limited solid solubility in Mg, such as a variety of transition elements, to improve mechanical properties [3]. Segregations in the mid-plane of the sheet, sticking of the sheet to the rolls, buckling, and deformation segregations constitute the common challenges of the TRC process [4] that have to be addressed by appropriate tuning of the TRC parameters.

Mg sheets produced by the TRC process typically exhibit textures with preferential orientation of the basal planes parallel to the rolling direction [5,6,7]. This results in anisotropic mechanical properties with respect to the direction of deformation. It is explained by the occurrence of the $\{10\bar{1}2\}\langle 10\bar{1}1\rangle$ twinning mode that contributes to the macroscopic strain only when stress is applied in compression parallel to the basal plane or in tension perpendicular to it [8]. In principle, the deformation twinning occurs as an accommodation mode in metals with low-symmetry crystal structures such as hcp, where the necessary five independent dislocation slip systems do not operate at room temperature. Therefore, the mechanical properties—ductility and yield stress—of magnesium alloys are considerably influenced by operable twinning modes [8]. 

Among different experimental techniques utilized for characterization of deformation mechanisms in hexagonal materials, the in-situ neutron diffraction (ND) method has been proven very efficient and conclusive [9,10]. Because neutrons have the long penetration length, they can provide microstructural information from the interior of the sample with a large volume. It has been shown that the integrated intensity of particular reflections is sensitive to the reorientation of the crystal lattice (e.g., due to twinning or deformation texture) during loading [11]. From the changes in the intensity of reflections, the relative volume of twins can be determined [12]. 

The acoustic emission (AE) technique is another in-situ method which has been widely used for characterization of deformation processes by detection of the transient elastic waves generated within the material due to sudden localized structural changes occurring during plastic deformation caused by dislocation motion and/or twinning. The AE parameters can be used to analyze collective dynamic processes that play a role during the plastic deformation of Mg alloys [13]. It should be noted that, while ND is sensitive to the twin volume, AE provides temporal information about twin nucleation. 

In the present work, we investigate the impact of the twin roll casting process and the subsequent heat treatment on the microstructure and the deformation mechanisms in ZEK100 magnesium alloys deformed at room temperature using ND and AE technique. The influence of the sample orientation and the microstructure evolution after heat treatment is discussed in detail. The alloy ZEK100 was developed recently as a potent structural [14] and bio-degradable [15] alloy with improved strength and corrosion resistance by alloying with zinc, zirconium and rare earth metals.

## 2. Materials and Methods

ZEK100 magnesium alloy, having a nominal composition of Mg-1.0 wt % Zn-0.3 wt % Zr-0.3 wt % Nd was used for experiments. The alloy was prepared by twin roll cast method. The rolls having 140 mm in diameter were made from a beryllium-copper alloy (Cu: 99.5 at %, Be: 0.5 at %). The melt containing pure Mg, Zn and Mg-15 at % Zr master alloy was melted at 700 °C and then rolled with a speed of 3 m/min. The final thickness of the sheets was 4 mm. The samples for the tensile test were prepared along both the rolling (RD) and the transverse (TD) direction. To study the influence of heat treatment, samples after T4 solution heat treatment (24 h at 413 °C) were also investigated. The deformation tests were carried out at a nominal strain rate of 10^−3^·s^−1^ on dog-bone-shaped flat samples, with the active part of 4 × 5 × 20 mm^3^. The deformation was stopped at several predefined strain levels (from 0.1% to 18%) for approximately 22 min to collect the diffraction data. The sample volume examined by neutrons was 4 × 4 × 4 mm^3^. The neutron experiments were performed on the ENGIN-X stress–strain diffractometer at the ISIS spallation neutron source in Chilton, UK. The ENGIN-X operates in time-of-flight (TOF) diffraction mode, using neutron pulses with a range of wavelengths 0.5–8.0 Å, which travel a distance of 50 m towards the sample before being elastically scattered. The instrument was equipped with a horizontal custom-built load frame by Instron with a ±100 kN capacity, mounted on the diffractometer with its loading axis at 45 degrees to the incident beam. Two detector banks: (i) axial; and (ii) radial, measure time-resolved diffraction patterns at fixed horizontal scattering angles centered on ±90°. 

In addition to the neutron measurements, the textures of both specimen types (as-rolled, heat treated) were investigated. The X-ray PANalytical XPert MRD diffractometer (PANanalytical, Almelo, The Netherlands) using Cu_Kα_ radiation with polycapillary optics in the primary beam was employed for acquiring the (0002) and $\left(10\bar{1}0\right)$ pole figures. The X-ray data were processed by MTEX library of program MatLab developed by MathWorks [16].

Concurrently with the diffraction experiments, the acoustic emission (AE) response was recorded using a PCI-2 acquisition board (Mistras Corp., Princeton, NJ, USA). The Micro-30S sensor giving a relatively flat rate response between 150–400 kHz and a 2/4/6-type 100–1200 kHz bandpass preamplifier with a total gain of 60 dB was used for AE recording.

The sample surfaces for microstructural investigation were ground on SiC papers (800, 1200, 2400 and 4000) and subsequently polished by diamond pastes down to # 0.25 μm grade. The polished surfaces for light optical microscopy (LOM, Olympus Europa, Hamburg, Germany) were etched with a solution of 50 mL of ethanol, 9 mL of water, 4 mL of acetic acid and 6 g of picric acid (98%) for 8 s. The specimen surface was finally ion beam polished on a Leica EM RES102 system (Leica Mikrosysteme, Wetzlar, Germany) before observation. The electron-backscatter diffraction (ESBD) observations were performed on FEI Quanta 200 FX scanning electron microscope (SEM, Thermo Fisher Scientific, Brno, Czech Republic). EBSD measurements were conducted at working distance of 13 mm by a step size of 1 μm with 15 kV acceleration voltage. The average confidence index of the measured maps is 0.75. The cleanup parameters for the evaluation of the EBSD data were set as follows: grain tolerance angle of 5 and minimum grains size of 5 (number of points). The exact sizes of the scanned areas are indicated in the captions of the particular Figures.

## 3. Results

### 3.1. Microstructural Observations

**Figure 1:**
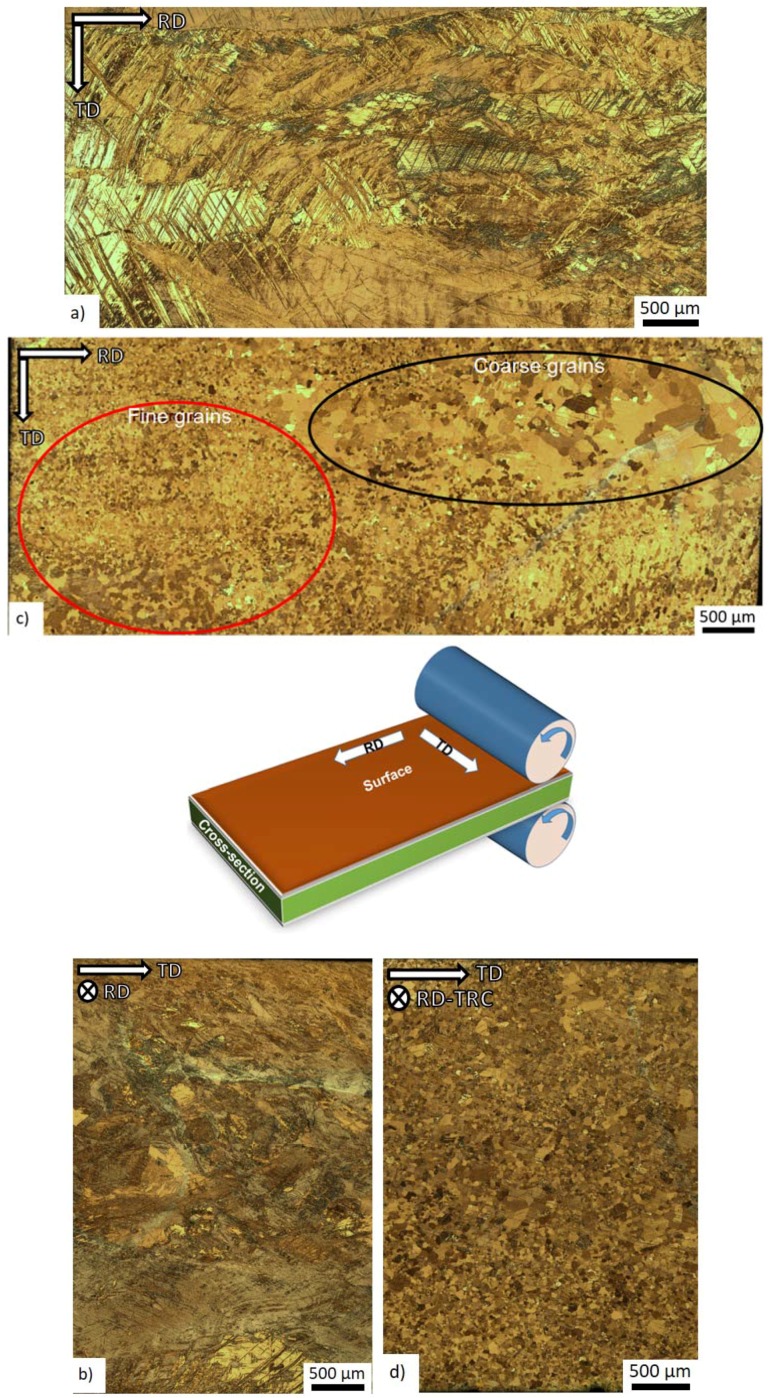
Optical micrographs of the *longitudinal surface* of: (**a**) *as-rolled*; and (**c**) *heat-treated* specimens; and micrographs of the *cross-section* of: (**b**) *as-rolled*; and (**d**) *heat-treated* specimens. The draw indicates the particular directions and sections, from which the micrographs were acquired. The red circles in Figure 1c indicate the fine grain areas, whereas the black circles the coarse grains.

**Figure 2:**
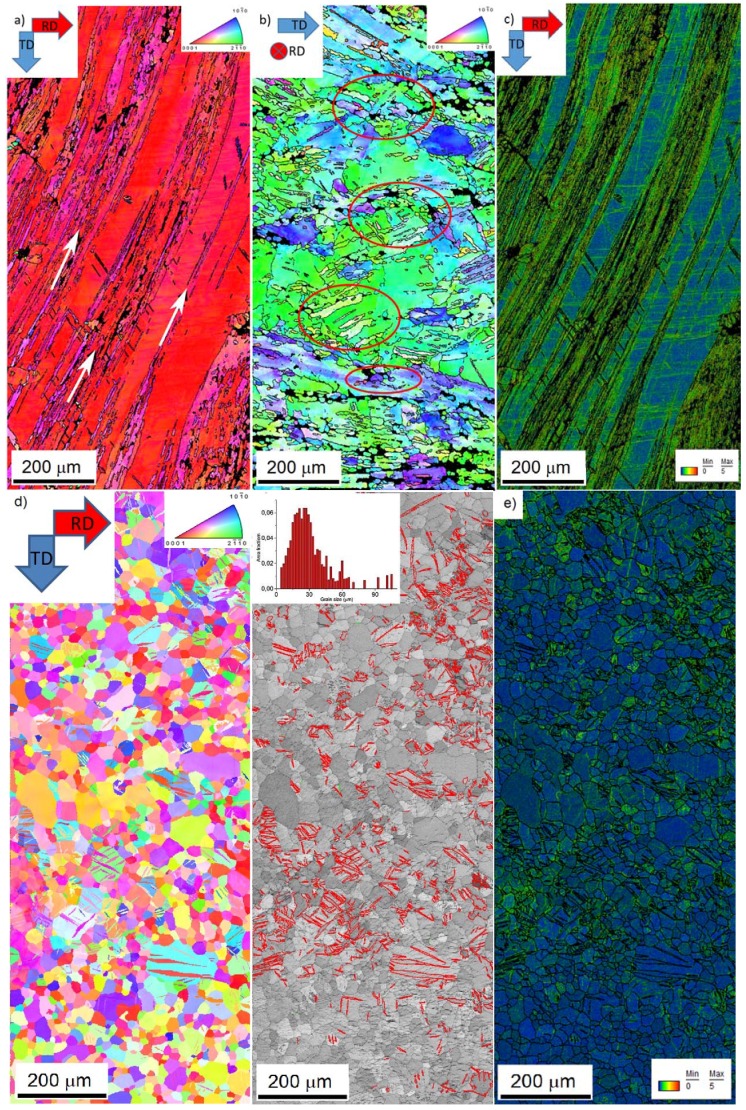
Orientation image maps (OIM) of the *as-rolled*: (**a**) surface (600 × 1300 µm)—the white arrows indicate the fine-grained strips; (**b**) cross-section (600 × 1300 µm); and (**c**) the corresponding Kernel Average Misorientation map (KAM) for the surface; (**d**) OIM map of the surface of the *heat-treated* specimen (550 × 1400 µm). On the corresponding image quality map, the extension twin boundaries (marked in red color) and the grains size distribution are indicated; (**e**) KAM map for the surface of the *heat-treated* specimen. The KAMs were calculated as the average misorientation between the data point and all of its first neighbors while excluding misorientations higher than 5°.

The initial microstructure of the both as-rolled and heat treated specimen is shown in Figure 1. In the as-rolled state, the microstructure is heterogeneous and complex (Figure 1a,b). On the surface of the sheet (perpendicular to the normal direction), preferentially coarse grains are present (Figure 1a). The EBSD map shows (Figure 2a) that the elongated grains have their basal planes oriented parallel to the rolling direction, and they are separated by “strips” containing the refined microstructure. The local misorientation distribution was examined using Kernel average misorientation maps (KAM). KAM map has a value for each pixel equal to the average disorientation that pixel has with its neighbors. In other words, the KAM calculates the average misorientation between the data point and all of its neighbors while excluding misorientations higher than 5°. We chose the threshold of 5° to eliminate any contribution from high angle boundaries. High values of KAM indicate orientation gradients, which are closely related to geometrically necessary dislocation (GND). Thus, the KAM values are good indicators of localized strain in materials [17].

It is obvious in Figure 2c that significant residual strain is present after the TRC process.

**Figure 3:**
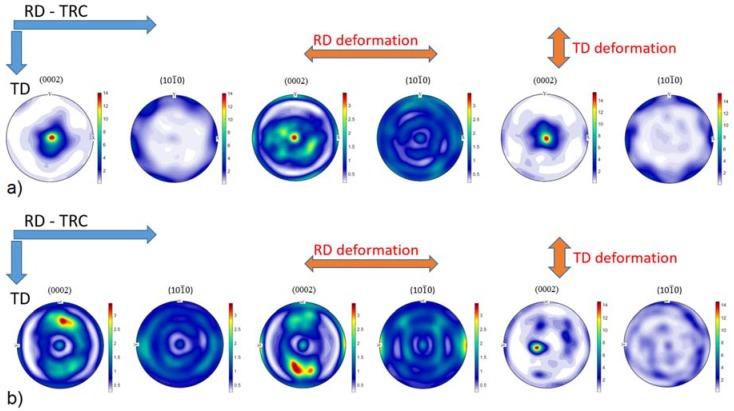
Evolution of (0001) and $\left(10\bar{1}0\right)$ pole figures for: (**a**) *as-rolled*; and (**b**) *heat treated* specimens as a function of the sample orientation with respect to the loading direction (measured by X-ray diffraction).

In the cross-section (perpendicular to rolling direction) of the initial state (Figure 1b), the microstructure depends on the distance from the surface. Close to the surface (top and bottom of Figure 1b), the microstructure is much finer than that in the middle section. The EBSD map shows that the share of the coarse grains prevails (Figure 2b—the thin black lines indicates the high angle boundaries (>15°). However, there is a larger portion of fine grains than that at the surface. The initial texture of the as-rolled material (Figure 3a) shows a slightly off-basal distribution.

During the solution heat treatment, recrystallization occurs. The microstructure at the surface still has a bimodal character, but many new fine grains were nucleated at the initial grain boundaries (Figure 1c). On the cross-section, the coarser grains are preferably concentrated in the middle section, whereas the areas on the top and bottom of Figure 1d (i.e., close to the surface) exhibit finer microstructure. The EBSD map (Figure 2d) of the cross-section shows a recrystallized microstructure. The average grain size estimated by linear intercept method is 14 µm, but the grain size distribution is rather wide. Particularly, the coarse grains contain extension twins, but many small twins can be observed throughout the whole structure. The KAM values are low (Figure 2e). However, some areas are not entirely relaxed. After the heat treatment, the (0002) pole figure of the sheet exhibits a broad tilt and split towards RD (Figure 3b). Further, the overall texture is weaker in comparison to the as-cast structure.

### 3.2. Mechanical Properties and Acoustic Emission (AE) Measurements

**Figure 4:**
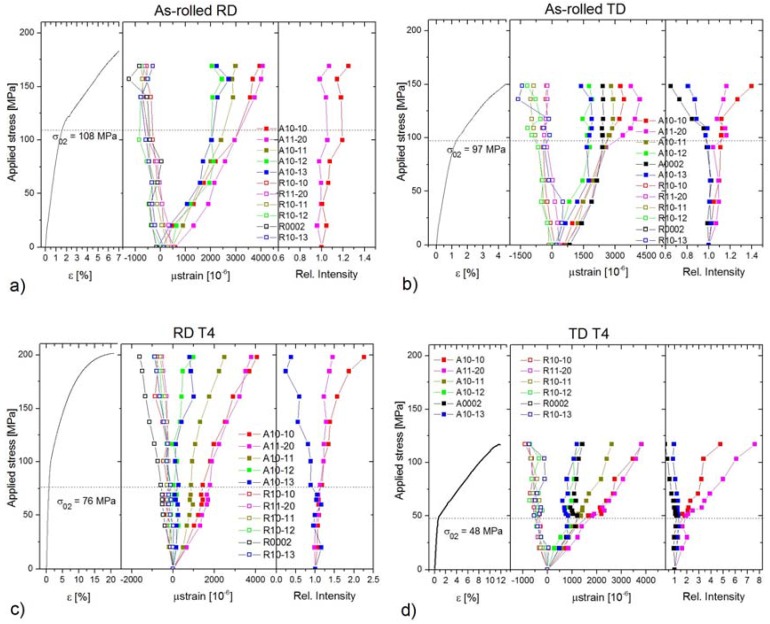
*Stress–strain curves and dependences of the lattice strains and integral intensities of* particular diffraction peaks in an axial and radial detector on the applied stress for *as-rolled* specimens tensioned in: (**a**) RD; and (**b**) TD directions; and *heat treated* (*T4*) specimens tensioned in: (**c**) RD; and (**d**) TD directions. The dash lines indicate the yield stress for the particular specimens.

**Figure 5:**
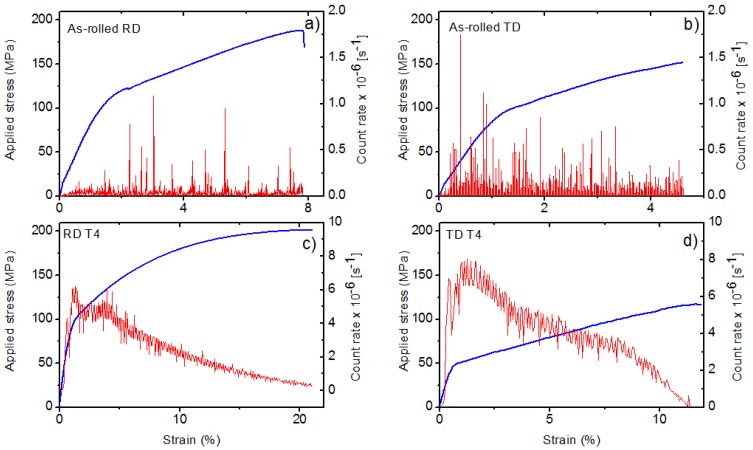
Acoustic emission response during tensile tests of the specimens in *as-rolled* condition along: (**a**) RD; and (**b**) TD directions; and after *heat treatment* along: (**c**) RD; and (**d**) TD directions.

The tensile deformation curves for both specimen conditions are shown in Figure 4. The yield strength is higher for the as-rolled specimens. This parameter is further influenced by the loading direction; in RD direction higher values were measured. The heat treatment enhances the elongation; it is almost six times higher after T4. The strain developments of the AE count rate (number of the crossing of the threshold level per second), measured during diffraction test, are shown in Figure 5. Several interesting features can be recognized on the AE curve. Firstly, AE appears at the very beginning of loading while the deformation is still macroscopically elastic. Secondly, the AE response is higher for TD specimens. Thirdly, the AE response of the heat-treated sample is approximately two orders higher near the yield stress. As shown by Friesel and Carpenter, there are two primary AE sources in magnesium alloys—dislocation slip and twinning [18]. In the case of dislocations, the AE signal is emitted by highly correlated motion of many dislocations. These so-called dislocation avalanches emerge as simultaneous breakaways of dislocations pinned at different obstacles (grain boundaries, solute atoms, precipitates, forest dislocations, etc.). Recent studies (e.g., [19]) indicate that this can take place already at low stresses, giving rise to localized micro-plasticity. Since the dislocation slip is simultaneously active in many grains, and the amplitude of the signal is rather low [20], the resultant dislocation AE signal is usually of a continuous noise-like type. In contrast, in the case of twinning, the AE detect the nucleation and propagation (i.e., growth of twin in length) stage of the mechanisms, which is an abrupt motion of strongly correlated twinning dislocations. Thus, the twinning emits burst signals. The amplitude of the transient AE twinning signal is directly proportional to the length of the twin [21]. In general, the AE events originated from twinning usually have higher amplitudes and power than those of the dislocation origin. As it was shown by several works [9,22,23], in magnesium alloys the peak around the yield point is connected with the synergic activity of non-basal dislocation slip and twinning. Our present results indicate that the twinning activity is higher in TD direction, particularly for the heat-treated specimen.

### 3.3. Neutron Diffraction (ND) Measurements

In the ND measurements, the evolution of six diffraction peaks, (0002), $\left\{10\bar{1}0\right\}$, $\left\{11\bar{2}0\right\}$, $\left\{10\bar{1}3\right\}$, $\left\{10\bar{1}1\right\}$ and $\left\{10\bar{1}2\right\}$, were investigated. The results can be used for estimation of the twinned volume and the activation stress of the particular deformation mechanisms. 

As shown first by Gharghouri [11], $\{10\bar{1}2\}\langle 10\bar{1}1\rangle$-type extension twinning causes intensity variation of the diffraction peaks. Owing to the used diffraction geometry, the reorientation of the lattice during the tension by 86.3° leads to the increase of $\left\{10\bar{1}0\right\}$ and $\left\{11\bar{2}0\right\}$ peaks, and, at the same time, to the decrease of (0002) and $\left\{10\bar{1}3\right\}$ ones. Thus, the (0002)–$\left\{10\bar{1}0\right\}$ and $\left\{10\bar{1}3\right\}$–$\left\{11\bar{2}0\right\}$ parent–twin systems characterize well the twinning activity. 

The activity of the particular deformation mechanisms can be deduced from the stress dependence of the lattice strains. It can be calculated using the Bragg equation:

(1) $$2d_{hkl}\sin\theta_{hkl}=\lambda$$

where $d_{hkl}$ is the spacing between adjacent (hkl) lattice planes. During straining, the $d_{hkl}$ value changes and the angular position of diffraction peaks shifts accordingly. Thus, the relative lattice strain can be calculated from the shift of the corresponding peak position as:

(2) $$\varepsilon_{hkl}=\frac{\left(d_{hkl}-d_{hkl}^{0}\right)}{d_{hkl}^{0}}=\frac{\Delta d_{hkl}}{d_{hkl}}=-\cot(\theta_{0,hkl})\Delta \theta_{hkl}\;(1)$$

where $d_{hkl}^{0}$ corresponds to the stress-free state when the lattice is unstrained. Determination of $d_{hkl}^{0}$ is fundamentally problematic because the ideal, stress-free state in the investigated polycrystalline is hard to reach. In our case, $d_{hkl}^{0}$ was considered as the initial position of the particular peak in the heat-treated material. As the EBSD measurements indicated, the residual stress level in this sample is low, so that our assumption seems to be reasonable. In the elastic regime, the lattice strain evolution follows the Hooke’s law. Thus, it is a linear function of the applied stress. The deviation of the lattice strain from the elastic response means the beginning of plastic deformation within a given grain family. 

According to this scheme, in Figure 4 we plotted the: (i) engineering stress–strain curves (left graph); (ii) the evolution of the elastic strains for both the axial and radial detector (middle graph); and (iii) the changes of the relative intensity in axial direction of particular “twinning related peaks” (right graph). 

Both as-rolled specimens exhibit a relatively large residual lattice strain (up to +800 µε) in the axial direction. This is in good agreement with the EBSD findings shown in Figure 2c, where the Kernel misorientation maps indicate large residual strains in the material (see the green color). To get a better overview of the stress evolution of the lattice strain, we indicated the experimentally determined yield stress values (*σ*_02_) by dash lines. 

In the as-rolled specimens, the lattice strain for the $\left\{10\bar{1}3\right\}$ peak deviates from the ideal elastic behavior around 55 MPa for both directions; this is far below the macroscopic yield point. The same is valid for the (0002) peak in TD (in RD this peak was too weak for fitting with sufficient confidence). This means that the extension twinning is active already in the early stage of deformation. Further, there is a relaxation of the lattice strain on the plane around 75 MPa for both specimens. In contrast, dislocation slip on the $\left\{10\bar{1}1\right\}$ plane appears to be more important in RD, where it activates around the macroscopic yield point. In TD, the onset of slip on these planes shifts to ~125 MPa. 

The characteristic feature of heat-treated specimens is the significant portion of micro-plasticity. In the case of RD, the $\left\{10\bar{1}3\right\}$–$\left\{11\bar{2}0\right\}$ parent–twin reflections, connected with the $\{10\bar{1}2\}\langle 10\bar{1}1\rangle$-type extension twinning lose their linearity around 40 MPa. The slope of the $\left\{11\bar{2}0\right\}$ curve increases, indicating its “soft”-orientation. Concurrently, the slope of the $\left\{10\bar{1}3\right\}$ curve decreases (“hard”-orientation). Thus, the coupling of these two planes is apparent. In RD, both the $\left\{10\bar{1}2\right\}$ and $\left\{10\bar{1}1\right\}$ reflections show the non-linear behavior below the yield point. In TD, only the $\left\{10\bar{1}2\right\}$ reflection exhibits the micro-yielding phenomenon.

The variation of the relative peak intensities, which is proportional to the twinned volume, strongly depends on the loading direction and heat treatment. The particular parent–twin reflections behave according to the theory: the intensities of the soft-oriented peaks $\left\{10\bar{1}0\right\}$ and $\left\{11\bar{2}0\right\}$ increase, whereas those of conjugated peaks decrease. In RD, the as-cast specimen exhibits only a small change in comparison to the heat-treated specimen. In TD, the twinned volume in the as-cast condition is a bit larger than that for RD. The twinning activity in the heat-treated specimen in TD is the largest among all specimens, as is evident from the right graph in Figure 4d. 

## 4. Discussion

The above-presented results indicate that the microstructure and the texture of the material in both conditions—as-rolled and heat-treated—and the loading direction collectively influence the active deformation mechanisms during straining. 

In the as-rolled state, the initial texture is similar to that observed for AZ31 sheets in the H24 temper condition (strain hardened and partially annealed) [1,24,25,26]. However, in our samples, peak intensity splitting of the (0001) pole toward both RD and TD occurs with a more pronounced deviation in TD (Figure 3). 

The deformation tests show a higher work-hardening capacity in RD. At the same time, the AE activity is lower for this specimen, which indicates a higher density of obstacles for dislocation movement [13]. 

After the deformation (Figure 3), the texture in RD decreases. In addition to the broader splitting of the basal pole, a new texture component appears around the rim of the (0002) pole figure. This behavior can be ascribed to the activity of non-basal <*a*>-dislocations [24,26,27]. Balik et al. [24] performed a thorough analysis of possible slip modes in rolled Mg sheets. These authors pointed out that the resulting texture is caused by the duplex prismatic <*a*>-slip. 

The activation of both basal and non-basal <*a*>-slip modes is also supported by the evolution in the lattice strain. As mentioned above, in RD the lattice strain on $\left\{10\bar{1}2\right\}$ planes begins to deviate from the ideal elastic response below the macroscopic yield. These planes have a high Schmid factor for the basal slip (0.43) [28]. The activation stress of this mechanism is higher than that measured for other magnesium alloys [29,30], but this difference can be substantiated by the heavily deformed initial microstructure and the relatively low number of grains favorably oriented for the basal slip. The relaxation of the $\left\{10\bar{1}1\right\}$ planes takes place in RD just below the macroscopic yield. Both basal (SF_basal_ = 0.36) and prismatic <*a*>-slip (SF_prism_ = 0.34) contribute to the plasticity on these planes. The importance of this mechanism in the macroscopic yield was proven both theoretically [29] by elastic plastic self-consistent (EPSC) modeling and experimentally by diffraction experiments [9]. The extension twinning as one deformation mechanism also cannot be neglected; the texture components around the rim of the (0001) pole figure indicate the activity of this twinning mode. However, the deformation in RD is rather controlled by slip. The EBSD measurements corroborate the diffraction experiments; in contrast to the initial state (Figure 2a), the OIM map of the as-rolled sample deformed in RD exhibits a larger variety of orientations in response to the dislocation slip activity. 

**Figure 6:**
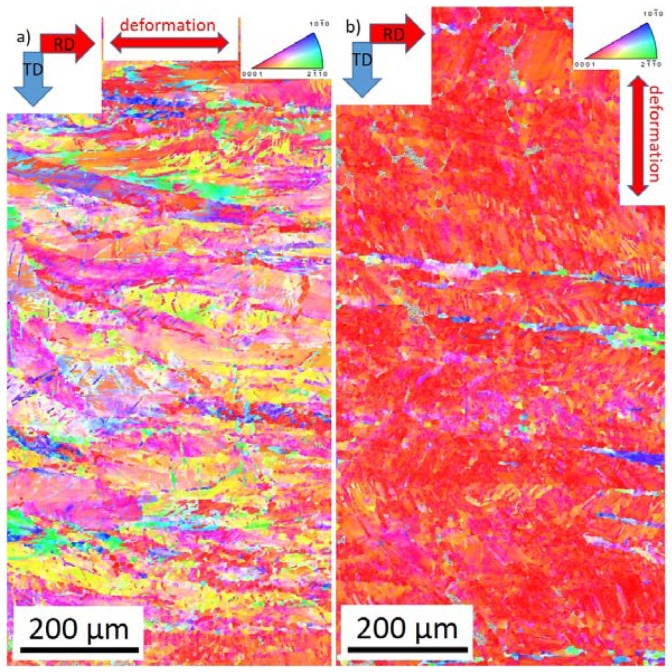
OIM maps of the longitudinal direction of the *as-rolled* specimens deformed in: (**a**) RD (550 × 1100 µm); and (**b**) TD directions (550 × 1100 µm).

In TD, the dislocation slip plays a minor role in plasticity. The onset of the slip on the $\left\{10\bar{1}1\right\}$ planes is far above the macroscopic yield stress (Figure 4b). Correspondingly, the texture after the deformation (Figure 3a and Figure 6b) is similar to that in the initial state. However, a new texture component in RD appears on the (0001) pole figure. This is clear evidence of activity of the $\{10\bar{1}2\}\langle 10\bar{1}1\rangle$-type extension twinning mechanism [26]. Even though the majority of grains are not favorably oriented for this deformation mode, it can be activated if the resolved shear on the twinning plane and in the twinning direction exceeds the critical stress (cf. numerical models of Jain and Wang [26,31]). The direct evidence for twinning in grains having low Schmid factor was obtained earlier in [9,32] by both neutron diffraction and EBSD. Finally, the more substantial change of the twinning-related diffraction peak intensities also supports the importance of the extension twinning in TD.

Both the AE and ND data are in good agreement with the texture measurements. The less intensive AE in RD can be elucidated as follows. Owing to the intensive slip activity, the dislocation density steeply increases during straining. This leads to decreasing of the mean free path of the dislocations and consequently to the lower AE response [13]. Further, the twin propagation in the presence of dislocation slip also slows down [21], which also leads to lowering the AE. In TD, the abundant burst AE corresponds well with the larger twinned volume estimated from ND. In Refs. [24,25], it was found that twinning is significant in the case of loading parallel to the direction of broadening of the intensity peak, i.e., twinning was more pronounced during tension along TD then RD in a sample with a basal texture and pronounced tilt of basal planes toward TD. This fact was also supported by the AE response and the microstructure observation (Figure 6).

**Figure 7:**
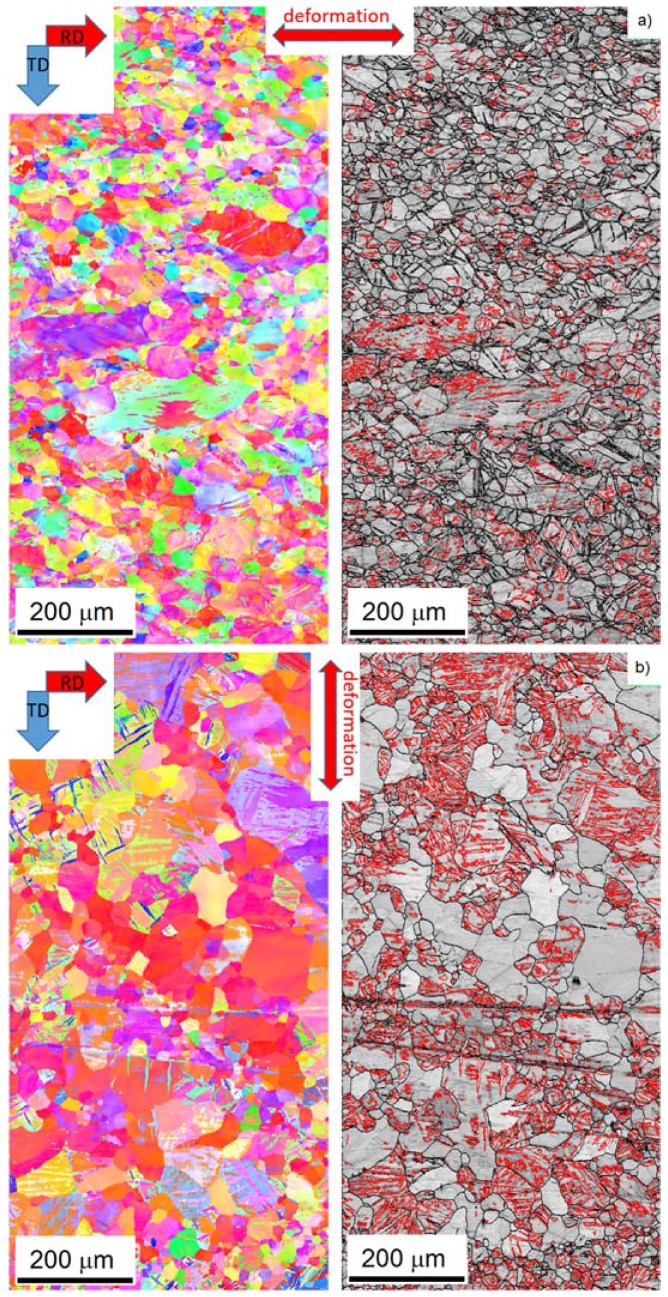
OIM maps of the surface of the *heat-treated* specimens, deformed in: (**a**) RD (550 × 1100 µm); and (**b**) TD directions (550 × 1100 µm). On the corresponding image quality maps, the extension twin boundaries are indicated in red color.

After the *heat treatment*, the texture changes. The tilt toward TD becomes more significant, similarly to that observed for the ZE10 sheet in O temper (heat treatment 16 h at 300 °C) condition [24]. After the deformation in RD picture remains with a slight increment in the angular spread toward TD (Figure 3a). The tension in TD causes a concentration of the intensity around the (0002) pole (Figure 3b). The similar results were observed for conventionally rolled alloys AZ31 and ZE10 after heat treatment for 8 h at 200 °C [24,25]. The difference in the texture evolution is given by the different conditions for activation of the extension twinning in the particular directions. In RD the substantial fraction of grains has their *c*-axis perpendicular to the loading direction, which means that these grains have the low Schmid factor for twinning. In contrast, in TD the conditions for twinning are much better. Accordingly, the measured ND intensity change in the TD direction is three times larger than that for RD. Furthermore, the AE response in the TD oriented specimens also exceeds that measured in RD. The EBSD maps (Figure 7) also show the higher twinned area for the TD sample. Thus, it is not surprising that owing to the profuse twinning, the yield stress in TD is only the half of that in RD (Figure 4c,d). The other consequence of the difference in twinning activity is that the non-basal <*a*> slip activates in RD at lower stresses than that in TD.

## 5. Conclusions

The combination of in-situ neutron diffraction and acoustic emission techniques with ex-situ texture measurement and microscopy obtained a complex characterization of the microstructural evolution and deformation mechanisms in twin roll cast Mg-Zn-Zr-Nd magnesium alloy. The results indicate that a strong basal texture forms during the TRC process with a slight off basal-character towards both RD and TD direction. In as-rolled condition the difference between the mechanical properties of the specimens deformed in RD and TD direction is small. However, in RD, higher work hardening is observed owing to the more intensive <*a*>-type dislocation activity. The heat treatment results in a recrystallization and significant anisotropy in the deformation properties. Owing to the easier activation of the extension twinning in TD given by texture, the yield stress in this direction is only half of that in RD.
